# Negative Relationship between Erythropoietin Dose and Blood Lead Level in Patients Undergoing Maintenance Hemodialysis

**DOI:** 10.1038/srep34313

**Published:** 2016-09-29

**Authors:** Wen-Hung Huang, Ching-Wei Hsu, Cheng-Hao Weng, Dan-Tzu Lin-Tan, Tzung-Hai Yen

**Affiliations:** 1 Department of Nephrology and Division of Clinical Toxicology and Toxicology Laboratory, Chang Gung Memorial Hospital, Linkou Medical Center, R.O.C., Taiwan; 2 Chang Gung University College of Medicine, Taoyuan, R.O.C., Taiwan

## Abstract

The adverse effects of increased blood lead levels have been well discussed. Several antioxidant agents have been reported to offer protection from lead toxicity and to reduce blood lead levels (BLL). Given that erythropoietin (EPO) also has antioxidant properties, the aim of this cross-sectional study was to assess the role of EPO and other clinical variables on BLL in hemodialysis (HD) patients. We recruited 931 maintenance hemodialysis (MHD) patients who had undergone HD for at least 6 months and who had ever received blood lead level (BLL) study. Use of erythropoiesis-stimulating agents followed the The National Kidney Foundation Kidney Disease Outcomes Quality Initiative (NKF KDOQI) Clinical Practice Guideline. We estimated demographic, hematological, nutritional, inflammatory, biochemical, and dialysis-related data based on this study. In the group with EPO, 7% had high BLL. In the group without EPO, 22% had high BLL. From the stepwise liner regression, urban areas, hemodialysis duration, and clearance of urea (KT/V_urea_) were positively associated with log BLL. In contrast, diabetes (DM), and monthly EPO dose were negatively associated with log BLL. This study showed that EPO dose might be negatively associated with blood lead levels in patients on maintenance hemodialysis.

The adverse effects of increased blood lead levels have been well investigated in several studies, including those focusing on general and chronic renal failure patients, or patients undergoing dialysis^1^,^2^,^3^,^4^,^5^,^6^,^7^. Several antioxidant agents have also been reported to confer protection against lead toxicity and to reduce blood lead levels (BLLs)^8^. Although the disadvantages and advantages of EPO have been thoroughly discussed^9^,^10^,^11^, EPO has antioxidant properties^12^,^13^,^14^,^15^. In 2007, Sakata *et al*. pointed out the negative association between BLLs and serum EPO concentrations in subjects with normal renal function^16^. In patients with chronic renal failure, Grzeszczak *et al*. reported that patients with EPO therapy had (nonsignificantly) lower blood lead levels than patients without it ref. 17. From the above studies, we can see a negative association between BLL and EPO. However, in Grzeszczak *et al*.’s^17^ study that did not take into account corrected related variables such as dialysis dose or living areas for BLL in advanced analysis, they did not find the statistic significant results. To the best of our knowledge, research on the relationship between use of EPO or EPO dose and BLL in HD patients is limited and the relationship remains obscure. The aim of this cross-sectional study was therefore to assess the role of EPO, EPO dose and other clinical variables on BLL in HD patients because of the abovementioned antioxidant properties of EPO.

## Results

### Study Population Characteristics

In total, this study comprised 931 MHD patients (470 men and 461 women) with a mean MHD duration of 6.71 ± 5.36 years. Table 1 lists the patient characteristics, including age, sex, and BMI, along with biological, hematological, and HD data. Of all the patients, 50.5% were male, 22% had a medical history of diabetes mellitus (DM), 4.6% had previous cardiosvascular diseases (CVDs), 18% were habitual tobacco users, 80.2% had an arteriovenous (AV) fistula, 92.3% had use of ESA, 11.3% had hepatitis B virus (HBV) infection, 18.4% had hepatitis C virus (HCV) infection, 8.5% of hemodialysis patients had high blood lead concentrations, 43.4% had high-normal blood lead concentrations, and 48.1% had low-normal blood lead concentrations.

**Figure 1:**
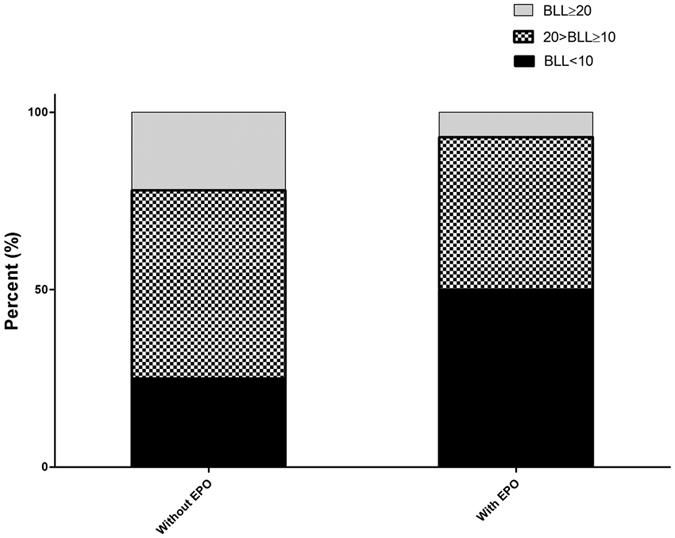
Comparison of percentage of low-normal, high-normal, and high BLL between patients with and without EPO use. In patients with EPO, 50% had low-normal BLL, 43% had high-normal BLL, and 7% had high BLL. In patients without EPO, 25% had low-normal BLL, 53% had high-normal BLL, and 22% had high BLL. *Low-normal BLL, BLL < 10 ug/dL; High-normal BLL, 20 ug/dL >BLL ≥ 10 ug/dL; High BLL, BLL ≥ 20 ug/dL.

Table 2 presents the subgroup analysis for patients with and without ESA. The patients without ESA had a longer HD duration (10.04 ± 6.80 vs. 6.43 ± 5.14 years, p < 0.001), higher body mass index (BMI) (23.17 ± 2.80 vs. 22.12 ± 3.21 kg/m^2^, p = 0.003) higher hemoglobin (Hb) level (12.33 ± 1.53 vs. 10.35 ± 1.21 g/dL, p = 0.02), higher intact-parathroid hormone (iPTH) level (188.6 vs. 123.8 pg/mL, p < 0.001), higher blood lead level (13.69 vs. 9.98 ug/dL, p < 0.001), higher calcium level (10.19 ± 1.00 vs. 9.90 ± 0.92 mg/dl, p = 0.022), higher phosphate level (5.18 ± 1.42 vs. 4.79 ± 1.35 mg/dl, p = 0.027), higher creatinine level (12.70 ± 2.16 vs. 10.72 ± 2.33 mg/dl, p < 0.001), lower ferritin level (62.1 vs. 329.8 μg/l, p < 0.001), lower KT/V_urea_ (1.68 ± 0.26 vs. 1.80 ± 0.32, p < 0.001), higher male sex prevalence (86.1% vs. 47.4%, p < 0.001), higher smoking prevalence (38.9% vs. 16.3%, p < 0.001), higher living in urban areas (40.3% vs. 16.2%, p < 0.001), and higher HCV prevalence (38.9% vs. 16.7%, p < 0.001). Patients with ESA also had lower prevalence of high and high-normal BLL, and higher low-normal BLL prevalence (Fig. 1).

**Figure 2:**
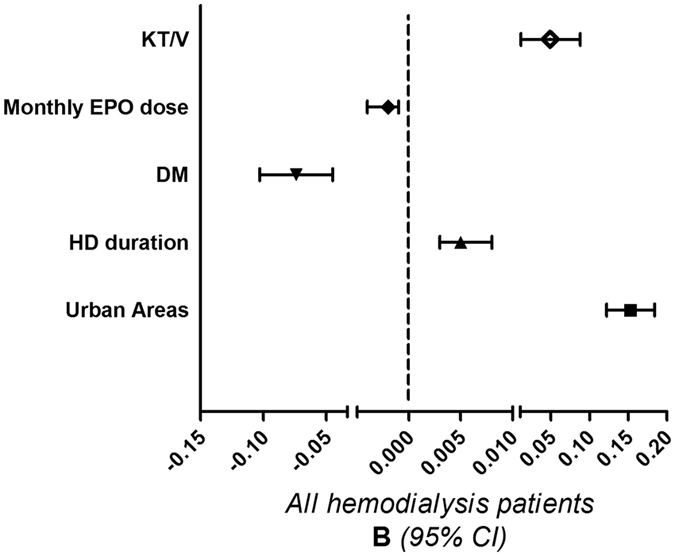
After adjustment for related variables (including body mass index, HCV, use of EPO, fistula as blood access, hemodiafiltration, hemoglobin, corrected calcium, log ferritin, log iPTH, and log hsCRP), urban areas (**B:** 0.153, 95% CI [0.122,0.184]), hemodialysis duration **(B:** 0.005, 95% CI [0.003,0.008]), and KT/V_**urea**_ **(B:** 0.049, 95% CI [0.011,0.088]) were positively associated with log BLL. However, DM **(B:** −0.074. 95% CI [−0.103,−0.045]), and monthly EPO dose **(B:** −0.002. 95% CI [−0.004,−0.001]) were negatively associated with log BLL. **B**: unstandardized coefficients 95% CI : 95% confidence interval.

To further clarify the factors associated with log lead level in our study patients, we used univariate and multivariate linear regression “stepwise” methods for analyses. Table 3 reveals the findings from the univariate linear regression: BMI (standardized coefficients (***β)***: −0.1, 95% confidence interval (CI) [−0.01, −0.002]), DM (***β***: −0.223, 95% CI [−0.138, −0.077]), erythropoietin dose U. kgw^−1^week^−1^ (***β***: −0.105, 95% CI [−+0.001, −0.0001]), use of EPO (***β***: −0.145, 95% CI [−0.156, −0.061]), log ferritin (***β***: −0.092, 95% CI [−0.065, −0.011]), and urban areas (***β***: 0.359, 95% CI [0.155, 0.217]) were negatively associated with log BLL, whereas HCV (***β***: 0.112, 95% CI [0.025, 0.091]), hemodialysis duration (***β***: 0.279, 95% CI [0.008, 0.013]), hemodiafiltration (HDF) (***β***: 0.153, 95% CI [0.043, 0.106]), Kt/V_urea_ (***β***: 0.16, 95% CI [0.06, 0.139]), Hb (***β***: 0.110, 95% CI [0.007, 0.026]), and log iPTH (***β***: 0.151, 95% CI [0.029, 0.071]) were positively associated with log BLL. An advanced multivariate linear regression analysis (Table 4) indicated that after adjustment for the studied variables, use of EPO (***β***: −0.064, 95% CI [−0.096,−0.0001]) was significantly correlated with log BLL. Table 5 also shows that after adjustment for the studied variables, EPO dose represented by U.kg^−1^week^−1^ (***β***: −0.112, 95% CI [−0.001,0.−0001]) was negatively associated with log BLL. In the advanced multivariate linear regression analysis, monthly EPO dose (**B**: −0.002, 95% CI [−0.004,−0.001]) was also associated with log BLL (Fig. 2).

## Discussion

In this study, we have shown that after adjustment for related variables, the level of blood lead showed a significantly negative association with the dose of EPO in maintenance HD patients.

To our knowledge, studies on the correlation between EPO and BLL in HD patients are few. Although our study is not the first to observe this correlation, we are the first to show a significantly negative correlation from an advanced analysis that included living environments and dialysis dose. In a cross-sectional study in London, Davenport *et al*.^18^ found that BLL was positively correlated with hemodialysis vintage, use of reverse osmosis water purification device unit, and that BLL was negatively correlated with residual urine output, with approximately 25.5% of patients having BLL >20 ug/dL. Colleoni *et al*.^7^ uncovered a positive correlation between BLL and PTH levels, but no correlation between BLL and HD duration; the authors also reported that the environmental risk factors (occupational exposure, tap water consumption and older houses) were associated with BLL in HD patients. Skarupskiene *et al*.^19^ also showed that HD duration >3 years was associated with elevated BLL. The findings from these abovementioned studies are mostly consistent with ours insofar as HD duration and environmental risk factors are important variables on BLL of HD patients.

It is natural to ask why KT/V_urea_ value was positively correlated with BBL. In general, the higher the KT/V_urea_, the more uremic toxin is removed from the body. However, higher dialysis dose did not confer any advantage against mortality^20^. It is interesting to note Skarupskiene’s^19^ finding that in a comparison of BLL before and after hemodialysis, BLL significantly increased after hemodialysis. Our finding and the above cited study may explain why there was no advantage of a higher KT/V_urea_ on mortality in hemodialysis patients^20^. However, further study on pre- and post-hemodialysis BLL are required to confirm this observation.

We know that erythropoietin is a hormone released from the kidney that stimulates erythropoiesis. However, it is not known why there is a negative correlation between monthly EPO dose and BLL in our study’s patients or in those of other studies^17^. In our knowledge, the mechanism on metabolism of heavy metals in hemodialysis are still obscure. Conversely, recent study in children on hemodialysis without aluminum (Al) containing phosphate binders, Manal *et al*.^21^. showed the positive association between BLL and EPO dosage. They also showed the positive association between blood Al levels and BLLs. We don’t know why the different results between our and Manal’s study. The number of studied population, age, dialysis duration, comorbidities, not including serum ferritin level and inflammation markers could be the reasons for the difference in both studies. However, in a previous study^22^, negative correlation between serum EPO and BLL was noted in pregnant women. Renal tubule toxicity of lead was suspected to the mechanism for reducing EPO generation^23^. However, in addition to stimulating erythropoiesis, the antioxidant property of EPO is another potential field of study. EPO was found to restore glutathione peroxidase activity^12^,^14^, reduce malondialdehyde concentrations^13^,^15^, and increase superoxide dismutase levels^12^. In addition, antioxidant nutrients and lead toxicity have been previously discussed in a review article that noted that vitamin C, vitamin E, vitamin B6, B-carotene, zinc, and selenium reduce lead-exposure toxicity^8^. Among these nutrients, vitamin C was observed to decrease the prevalence of elevated BLL^24^,^25^ and as a result has been the agent receiving the most attention for lead toxicity. In lead-poisoned rats, ascorbic acid was reported to have the equivalent chelating property of EDTA^26^. Although Dhawan *et al*. reported that vitamin C might increase lead elimination from urine in rats^27^, in Dawson *et al*.’s^24^ human study, they found that supplementation with 1000 mg, not 200 mg vitamin C per day results in a decrease of BLLs in general population, which is possibly due to a reduction in the intestinal absorption of lead. In our study, we reported that residual renal function was not associated with BLLs, and that the dose of EPO was negatively associated with BLL. From the above cited studies, it is clear that EPO has the property of antioxidant that may possibly reduce lead absorption from the intestine, and that this effect may be dose-dependent. However, further research is necessary to confirm the associations found in the present study.

It is interesting that the DM condition was negatively associated with BLLs in our studied subjects. Similarly, Forte reported patients with DM had a lower level of blood lead than the controls^28^. Taking Forte’s and our findings together, we speculate that HD patients with DM condition might have a greater chance of DM enteropathy inducing poor appetite and bowel functioning, which might reduce food intake, digestion and intestinal lead absorption.

This study has some limitations. First, this study showed the correlation between blood lead levels and EPO dose of HD patients in a cross-sectional design study. Therefore, further prospective studies are worth further evaluation whether use of EPO will reduce the blood lead levels of patients with MHD. Second, the number of patients with ESA were larger than patients without it (858:73). In our knowledge, most of HD patients must receive ESA to keep hematogenesis. Hence, the rate of above mention is reasonable. Third, we did not offer the information of usage of phosphate binders (including aluminum containing phosphate binders). After all, blood Al level may be a positive factor associated with BLL^21^. However, in our study, we did not find the significant correlation between blood Al and Pb levels (Table 3). Further advanced study could be designed for the association between usage of aluminum containing phosphate binder and BLLs. Fourth, we found that living in urban areas was a risk factor with elevated blood lead levels. We know that, comparing with rural areas, there are more people, transportation, factories and air pollution in urban areas. Hence, further advanced study for investigating above issue on blood heavy metals in HD patients is needed.

## Conclusion

This is the first study to demonstrate that EPO dose may be significantly associated with blood lead levels in patients on maintenance hemodialysis. Further studies are required to clarify these observations.

## Methods

This study protocol was approved by the Institutional Review Board Committee of Chang Gung Memorial Hospital. Written informed consent was obtained from all patients. All medical records, including medical history, laboratory data, and inclusion and exclusion criteria, were reviewed by senior nephrologists during the study period. All patient information was protected and available to only the investigators. And all experiments protocols were conducted according to the Strengthening the Reporting of Observational Studies in Epidemiology guidelines.

### Patients

**Figure 3:**
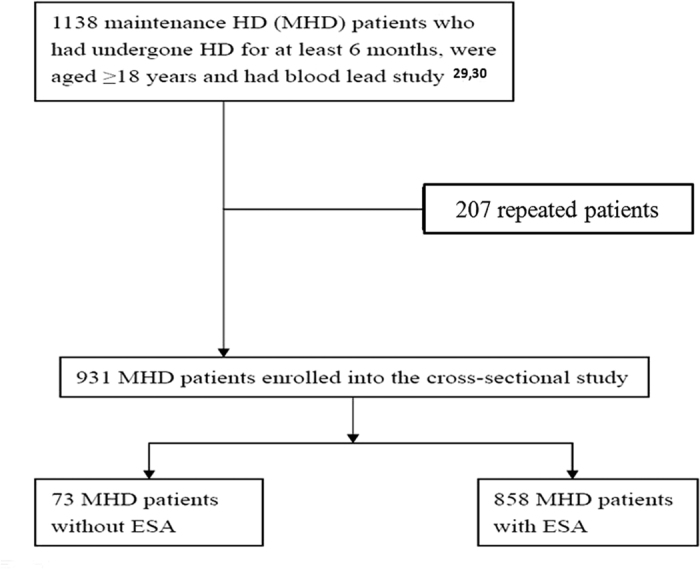
Flow chart shows enrollment of patients.

Patients were recruited from the HD centers of the Chang Gung Memorial Hospital branches in Linkou, Taipei, and Taoyuan. Only MHD patients who had undergone HD for at least 6 months, were aged ≥18 years and had blood lead study were enrolled^29^,^30^. Figure 3 showed the flow chart of enrollment of studied patients. Patients with malignancies or infectious diseases or who had been hospitalized or had undergone surgery within the previous 3 months were excluded. Diabetes mellitus was identified according to either a physician’s diagnosis, antidiabetic drug treatment, or 2 subsequent analyses demonstrating fasting blood glucose levels of >126 mg/dL. Most patients underwent 4 h of HD 3 times a week. HD was performed using single-use hollow-fiber dialyzers equipped with modified cellulose, polyamide, or polysulfone membranes. The dialysate used in all cases had a standard ionic composition with a bicarbonate-based buffer. Regarding HDF, patients who had undergone HDF 3 times a week for ≥3 months were enrolled. We evaluated the prevalence of CVDs, including cerebrovascular disease, coronary artery disease, congestive heart failure, and peripheral vascular disease, in the patients. Hypertension was defined as the regular use of antihypertensive drugs for controlling blood pressure or at least 2 blood pressure measurements of >140/90 mm Hg. Smoking behavior was also analyzed.

### Use of Erythropoiesis-Stimulating Agents (ESA)

Use of ESA followed the NKF KDOQI Clinical Practice Guideline^31^. All the patients in this study received epoetin beta (EPO-β, Roche, Basel, Switzerland).

### Laboratory, Nutritional, and Inflammatory Parameters

All blood samples were obtained from the arterial end of the vascular access immediately after the initial 2-day interval for HD and were then centrifuged and stored at −80 °C until use. Serum creatinine levels, nPCRs, and serum albumin levels were assayed and recorded as nutritional markers. High-sensitivity C-reactive protein (hsCRP) levels were measured as the indices of inflammation. Serum hsCRP level was measured using immunonephelometry (Nanopia CRP; Daiichi Inc., Tokyo, Japan). The lowest detection limit was <0.15 mg/L. All other biochemical parameters were measured using the standard laboratory approach with an automatic analyzer. In the HD patients, the dialyzer clearance of urea was measured using the method described by Daugirdas and was expressed as Kt/V_urea_^32^. The nPCR of the HD patients was calculated using validated equations and was normalized to their body weight^33^. The serum calcium level was corrected using the serum albumin level with the following formula: corrected calcium level (mg/dL) = serum calcium level + 0.8 × (4.0–serum albumin level). Nonanuria was defined as daily urine output of ≥100 mL.

### Measurement of Blood Lead Levels and Lead Levels in Water and Dialysate

To exclude the possibility that patients on maintenance hemodialysis were exposed to lead through the contamination of water or dialysate during hemodialysis, we collected at least 2 samples of water and dialysate from the outlets of the reverse osmosis systems and the inlets of the dialysate of the dialyzers in lead-free plastic bottles from each hemodialysis center^29^. Lead levels in water and dialysate were less than 2 ug/L. Blood lead levels were measured using a method described previously^29^,^30^,^34^. Lead levels were measured using an electrothermal atomic-absorption spectrometer (SpectrAA-200Z; Varian, Lexington, MA, USA) with Zeeman background correction and a L’vov platform. A certified commercially prepared product (Seronorm Trace Elements; Sero AS, Billingstads, Norway) was used to determine intra-batch accuracy and confirm the inter-batch standardization. The coefficient of variation for lead measurement was ≦5.0%. External quality control was maintained by participating in the National Quality Control Program conducted by the government. Blood lead levels of each patient were measured 2 times with a 3-month interval. The grade of BLL was defined as: Low-normal BLL, BLL <10 ug/dL; High-normal BLL, 20 ug/dL > BLL ≥ 10 ug/dL; High BLL, BLL ≥ 20 ug/dL^18^,^30^.

### Statistical Analysis

Data were analyzed using SPSS version 12.0 for Windows 95 (SPSS Inc, Chicago, IL, USA). The normal distribution of variables was analyzed using the Kolmogorov–Smirnov test. A P value of >0.05 was considered to indicate normal distribution. Data are expressed in terms of median and interquartile range in non-normal distribution variables and as mean ± standard deviation in normal distribution variables and categorical variables as numbers or percentages. Chi-square or Fisher exact tests were used for analyzing the correlation among categorical variables. Comparisons between two groups were performed using the Mann–Whitney U test and Student’s t-test. The data on hsCRP, iPTH, BLL, blood aluminum (Al) levels and ferritin levels were log-transformed for analysis.

To evaluate the variables related to BLL, univariate and multivariate (stepwise method) linear regression analyses were performed to assess the standardized coefficients (*β*) or unstandardized coefficients (B) and 95% confidence interval (CI) for the baseline variables that included age, male sex, BMI, smoking status, diabetes mellitus, hypertension, previous CVD, HBV infection, HCV infection, haemodialysis duration, fistula for blood access, HDF, use of EPO, monthly EPO dose, EPO dose represented as U.kg^−1^week^−1^, Kt/V_urea_ Daugirdes, nPCR. non-anuria status, Hb levels, serum albumin levels, serum creatinine levels, corrected-calcium levels, inorganic phosphate levels, log ferritin levels, log iPTH levels, log hsCRP levels, log Al levels, cholesterol levels, triglyceride levels, and urban areas (variables with p value < 0.1 in univariate linear regression were selected into multivariate linear regression). All the nominal variables in the logistic regression were transformed into dummy coding. Missing data were removed using listwise deletion. The level of significance was set at p < 0.05.

## Additional Information

**How to cite this article**: Huang, W.-H. *et al*. Negative Relationship between Erythropoietin Dose and Blood Lead Level in Patients Undergoing Maintenance Hemodialysis. *Sci. Rep.* **6**, 34313; doi: 10.1038/srep34313 (2016).

## Figures and Tables

**Table 1:** Characteristics of studied 931 MHD patients.

Characteristics	Total (931) Mean ± SD/Median (Range)
*Demographics*	
Age (years)	56.01 ± 13.57
Male sex (Yes), pt. No.	470 (50.5%)
Body mass index (kg/m^2^)	22.20 ± 3.19
Smoking (Yes), pt. No.	168(18%)
Urban areas	168(18%)
*Co-Morbidity*	
Diabetes mellitus (Yes), pt. No.	205(22%)
Hypertension (Yes), pt. No.	361(38.8%)
Previous CVD (Yes), pt. No.	43(4.6%)
HBV (Yes), pt. No.	105(11.3%)
HCV (Yes), pt. No.	171(18.4%)
*Dialysis Related Data*	
Haemodialysis duration (y)	6.71 ± 5.36
Erythropoietin (U/kg/week)	74.09 ± 46.97
Use of ESA	858 (92.3%)
Fistula as blood access (Yes), pt. No.	747(80.2%)
Hemodiafiltration (Yes), pt. No.	196(21.1%)
Kt/V _urea_ Daugirdes	1.79 ± 0.32
nPCR (g/kg/day)	1.18 ± 0.26
Residual daily urine of >100 ml, pt. No.	195(20.9%)
*Biochemical Data*	
Haemoglobin (g/dl)	10.50 ± 1.34
Albumin (g/dl)	4.07 ± 0.34
Creatinine (mg/dl)	10.87 ± 2.38
Ferritin (μg/l)*	305.8(130.2,506.55)
Corrected-calcium (mg/dl)	9.92 ± 0.92
Phosphate (mg/dl)	4.82 ± 1.36
Intact parathyroid hormone (pg/ml)*	129.1(52.6, 307.7)
hsCRP (mg/l)*	2.85(1.35, 6.86)
Blood aluminium (ug/dl)	0.9 (0.6, 1.4)
*Cardiovascular Risks*	
Cholesterol (mg/dl)	171.89 ± 37.40
Triglyceride (mg/dl)	164.13 ± 117.30
Blood lead (Pb) (ug/dL)*	10.22(7.22,13.96)
Low-normal blood lead level, pt No.	448(48.1%)
High-normal blood lead level, pt No.	404(43.4%)
High blood lead level, pt No.	79(8.5%)

Abbreviations: CVD: cardiovascular disease, HBV: hepatitis B virus infection, HCV: hepatitis C virus infection, nPCR, normalized protein catabolic rate, hsCRP = high-sensitivity C-reactive protein, LDL = low density lipoprotein, Kt/V_urea_ = dialysis clearance of urea, BLL = blood lead levels, Low-normal BLL, BLL < 10 ug/dL; High-normal BLL, 20 ug/dL >BLL ≥ 10 ug/dL; High BLL, BLL ≥ 20 ug/dL.

^*^Non-normal distribution data are presented as median (interquartile range).

**Table 2:** Comparison between MHD patients with EPO and without EPO.

Characteristics	Without EPO (73)	With EPO (858)	P
Age (y)	54.40 ± 11.00	56.12 ± 13.75	0.3
Male sex	63(86.1%)	407(47.4%)	<0.001
Body mass index (kg/m^2^)	23.17 ± 2.80	22.12 ± 3.21	0.003
Smoking (Yes)	28(38.9%)	140(16.3%)	<0.001
Diabetes mellitus (Yes)	12(15.3%)	193(22.5%)	0.18
Hypertension (Yes)	30(41.7%)	331(38.6%)	0.61
Previous CVD (Yes)	3(4.2%)	40(4.7%)	0.99
HBV (Yes)	12(16.7%)	93(10.8%)	0.17
HCV (Yes)	28(38.9%)	143(16.7%)	<0.001
Haemodialysis duration (year)	10.04 ± 6.80	6.43 ± 5.14	<0.001
Fistula as blood access (Yes)	59(80.6%)	688(80.2%)	0.99
Hemodiafiltration (Yes)	21(29.2%)	175(20.4%)	0.09
Kt/V Daugirdes	1.68 ± 0.26	1.80 ± 0.32	<0.001
nPCR (g/kg/day)	1.18 ± 0.22	1.18 ± 0.26	0.88
Non-Anuria (>100 cc/day)	9(12.5%)	186(21.7%)	0.07
Haemoglobin (g/dl)	12.33 ± 1.53	10.35 ± 1.21	<0.001
Albumin (g/dl)	4.01 ± 0.29	4.07 ± 0.34	0.12
Creatinine (mg/dl)	12.70 ± 2.16	10.72 ± 2.33	<0.001
Ferritin (μg/l)*	62.1(26.82, 150.1)	329.8(167.32, 519.85)	<0.001
Corrected-calcium (mg/dl)	10.19 ± 1.00	9.90 ± 0.92	0.022
Phosphate (mg/dl)	5.18 ± 1.42	4.79 ± 1.35	0.027
Intact parathyroid hormone (pg/ml)*	188.6(84.75, 404.17)	123.8(50.8, 293.67)	0.012
hsCRP (mg/l)*	2.21(1.39, 7.01)	2.89(1.34, 6.84)	0.74
Cholesterol (mg/dl)	165.18 ± 37.65	172.51 ± 37.33	0.116
Triglyceride (mg/dl)	166.24 ± 110.10	164.05 ± 117.98	0.87
Blood lead (Pb) (ug/dL)*	13.69(10.49, 19.37)	9.98(7.15, 13.68)	<0.001
Blood aluminum (ug/dl)*	1.0(0.75, 1.70)	0.9(0.57, 1.40)	0.061
Urban areas	29(40.3%)	139(16.2%)	<0.001

Abbreviations: EPO: Erythropoietin, MHD: maintenance haemodialysis, nPCR, normalized protein catabolic rate, HBV: hepatitis B virus infection, HCV:hepatitis C virus infection. hsCRP = high-sensitivity C-reactive protein, Kt/V urea = dialysis clearance of urea, Note: A P of  < 0.05 represents significant variance between the groups.

^*^Non-normal distribution data are presented as median (interquartile range).

**Table 3:** Univariate Linear Regression Analysis between log Pb and clinical variables in MHD Patients.

Characteristics	Univariate Linear Regression	P value
*Variables*	Standardized Coefficients (β) 95% confidence Intervals (CI)	
Age (years)	−0.025(−0.001, 0.001)	0.440
Male sex	0.05(−0.006, 0.046)	0.126
Body mass index (kg/m^2^)	−0.1(−0.01,−0.002)	0.002
Smoking (Yes)	0.015(−0.025, 0.042)	0.638
Diabetes mellitus (Yes)	−0.223(−0.138, −0.077)	<0.001
Hypertension (Yes)	−0.05(−0.047, 0.006)	0.129
Previous CVD (Yes)	−0.011(−0.071, 0.051)	0.743
HBV (Yes)	0.049(−0.01, 0.071)	0.137
HCV (Yes)	0.112(0.025, 0.091)	0.001
Haemodialysis duration (years)	0.279(0.008, 0.013)	<0.001
Erythropoietin (U/kg/week)	−0.105(−0.001, −0.0001)	0.001
Use of EPO (Yes)	−0.145(−0.156, −0.061)	<0.001
Fistula as blood access (Yes)	0.059(−0.003, 0.062)	0.073
Hemodiafiltration (Yes)	0.153(0.043, 0.106)	<0.001
Kt/V_urea_ (Daugirdes)	0.16(0.06, 0.139)	<0.001
nPCR (g/kg/day)	−0.004(−0.051, 0.046)	0.915
Non-Anuria	−0.039(−0.051, 0.013)	0.238
Haemoglobin (g/dl)	0.110(0.007, 0.026)	0.001
Albumin (g/dl)	−0.03(−0.055, 0.020)	0.361
Creatinine (mg/dl)	0.022(−0.004, 0.007)	0.510
C-Ca (mg/dl)	0.054(−0.002, 0.026)	0.097
Phosphate (mg/dl)	0.0001(−0.009, 0.009)	0.996
Log Ferritin	−0.092(−0.065, −0.011)	0.005
Log iPTH	0.151(0.029, 0.071)	<0.001
Log hsCRP	−0.057(−0.048, 0.003)	0.086
Log Al	0.048(−0.009, 0.064)	0.142
Cholesterol (mg/dl)	0.003(−0.0001, 0.0001)	0.933
Triglyceride (mg/dl)	−0.047(−0.0001, 0.0001)	0.154
Urban areas	0.359(0.155, 0.217)	<0.001

Abbreviations: Pb: lead, MHD: maintenance haemodialysis, CTS, carpal tunnel syndrome, nPCR, normalized protein catabolic rate, BMI = body mass index, HBV: hepatitis B virus infection. HCV: hepatitis C virus infection, DM = diabetes mellitus, CKD = chronic kidney disease, hsCRP = high-sensitivity C-reactive protein, iPTH = intact parathyroidhormone, Kt/V urea = dialysis clearance of urea, C-Ca = corrected calcium. Al:aluminum.

**Table 4:** Multivariate Linear Regression Analysis between log Pb and clinical variables in MHD Patients.

Variables*	Multivariate linear regression standardized coefficients (β) 95% confidence Intervals (CI)	P value
Diabetes mellitus (Yes)	−0.145(−0.099, −0.041)	<0.001
Haemodialysis duration (years)	0.140(0.003, 0.008)	<0.001
Use of EPO (Yes)	−0.064(−0.096, −0.0001)	0.049
Kt/V_urea_ (Daugirdes)	0.083(0.013, 0.09)	0.009
Log Ferritin	−0.068(−0.054, −0.002)	0.034
Urban areas	0.303(0.124, 0.186)	<0.001

^*^After adjustment for body mass index, HCV, fistula as blood access, hemodiafiltration, haemoglobin, corrected calcium, Log iPTH, and Log hsCRP.

**Table 5:** Multivariate Linear Regression Analysis between log Pb and clinical variables in MHD Patients.

Variables*	Multivariate linear regression standardized coefficients (β) 95% confidence Intervals (CI)	P value
Diabetes mellitus (Yes)	−0.154(−0.104, −0.045)	<0.001
Haemodialysis duration (years)	0.149(0.003, 0.008)	<0.001
Erythropoietin (U/kg/week)	−0.112(−0.001, 0.−0001)	<0.001
Kt/V_urea_ (Daugirdes)	0.098(0.021, 0.101)	0.003
Urban areas	0.296(0.121, 0.182)	<0.001

^*^After adjustment for body mass index, HCV, use of EPO, fistula as blood access, hemodiafiltration, haemoglobin, corrected calcium, Log ferritine, Log iPTH, and Log hsCRP.
